# Under the background of the new global definition of ARDS: an interpretable machine learning approach for predicting 28-day ICU mortality in patients with sepsis complicated by ARDS

**DOI:** 10.3389/fphys.2025.1617196

**Published:** 2025-09-19

**Authors:** Peijie Zhang, Shuo Yuan, Shuzhan Zhang, Zhiheng Yuan, Zi Ye, Lanxin Lv, Hongning Yang, Hui Peng, Haiquan Li, Ningjun Zhao

**Affiliations:** ^1^ Department of Emergency Medicine, The Affiliated Hospital of Xuzhou Medical University, Xuzhou, Jiangsu, China; ^2^ Xuzhou Key Laboratory of Emergency Medicine, The Affiliated Hospital of Xuzhou Medical University, Xuzhou, Jiangsu, China; ^3^ Department of Emergency Medicine, Feng Xian People’s Hospital, Xuzhou, Jiangsu, China; ^4^ Department of Respiratory and Critical Care Medicine, Second Affiliated Hospital of Xuzhou Medical University, Xuzhou, Jiangsu, China

**Keywords:** sepsis, ARDS, machine learning, 28-day, ICU mortality

## Abstract

**Background:**

Acute respiratory distress syndrome (ARDS) is a prevalent clinical complication among patients with sepsis, characterized by high incidence and mortality rates. The definition of ARDS has evolved over time, with the new global definition introducing significant updates to its diagnosis and treatment. Our objective is to develop and validate an interpretable prediction model for the prognosis of sepsis patients complicated by ARDS, utilizing machine learning techniques in accordance with the new global definition.

**Methods:**

This study extracted data from the MIMIC database (version MIMIC-IV 2.2) to create the training set for our model. For external validation, this study used data from sepsis patients complicated by ARDS who met the new global definition of ARDS, sourced from the Affiliated Hospital of Xuzhou Medical University. Lasso regression with cross-validation was used to identify key predictors of patient prognosis. Subsequently, this study established models to predict the 28-day prognosis following ICU admission using various machine learning algorithms, including logistic regression, random forest, decision tree, support vector machine classifier, LightGBM, XGBoost, AdaBoost, and multi-layer perceptron (MLP). Model performance was assessed using ROC curves, clinical decision curves (DCA), and calibration curves, while SHAP values were utilized to interpret the machine learning models.

**Results:**

A total of 905 patients with sepsis complicated by ARDS were included in our analysis, leading to the selection of 15 key variables for model development. Based on the AUC of the ROC curve, as well as DCA and calibration curve results from the training set, the support vector classifier (SVC) model demonstrated strong performance, achieving an average AUC of 0.792 in the internal validation set and 0.816 in the external validation set.

**Conclusion:**

The application of machine learning methodologies to construct prognostic prediction models for sepsis patients complicated by ARDS, informed by the new global definition, proves to be reliable. This approach can assist clinicians in developing personalized treatment strategies for affected patients.

## 1 Introduction

Sepsis is a systemic inflammatory response syndrome typically triggered by infection. The persistent systemic inflammatory response and the imbalance of immune regulatory mechanisms represent the core pathological and physiological processes underlying sepsis, often resulting in severe multi-organ dysfunction and posing a significant threat to life (Singer et al., 2016). A recent study examining patients with sepsis and septic shock from 2009 to 2019 indicated that conservatively, there are over 30 million new cases of sepsis globally each year, with approximately 6 million patients succumbing to sepsis or septic shock (Bauer et al., 2020). Additionally, a cross-sectional study conducted in China revealed that patients admitted to the ICU with sepsis had a 90-day mortality rate of around 35.5% (Xie et al., 2020).

The lungs are the first and most commonly affected organ in the progression of sepsis. Patients with sepsis may develop acute lung injury (ALI) or even acute respiratory distress syndrome (ARDS), which is characterized by refractory hypoxemia and respiratory distress. ARDS is a serious and potentially fatal respiratory failure marked by increased permeability of alveolar capillary membranes due to various direct or indirect injurious factors, resulting in edema in the alveoli and interstitial, as well as alveolar hemorrhage and the formation of hyaline membranes. These changes ultimately lead to hypoxemia and respiratory distress.

The combination of sepsis and ARDS is thought to be linked to mechanisms such as systemic inflammatory cytokine storms triggered by infection (Zhu et al., 2022), monocyte-macrophage activation (Lv and Liang, 2025), oxidative stress (Liu Y. et al., 2021), and a reduction in pulmonary surfactant or alterations in its composition (Whitsett et al., 2015), all of which may result in irreversible lung damage.

The clinical definition of ARDS has undergone several revisions, with the Berlin definition published in 2012 playing a pivotal role in clinical diagnosis and management. This definition emphasizes mechanical ventilation, the oxygenation index (PaO2/FiO2 ratio), and pulmonary imaging as essential parameters for diagnosing ARDS and assessing its severity (Ranieri et al., 2012). However, over the past decade, numerous medical professionals have identified limitations within the Berlin definition during clinical practice. In response, 32 critical care experts from around the world jointly published a new global definition of ARDS in May 2023. This updated definition broadens the diagnostic criteria for ARDS in patients receiving non-invasive ventilation and high-flow oxygen therapy (HFNO). It identifies non-invasive pulse oximetry, specifically the SpO2/FiO2 index, as a crucial indicator for diagnosing ARDS, replacing the traditional oxygenation index that relies on arterial blood gas analysis. Furthermore, pulmonary ultrasound has also been added as a supplementary tool for pulmonary imaging diagnosis (Matthay et al., 2024).

This new global definition significantly expands the application of ARDS clinical criteria, implementing important updates in diagnostic standards, scope, and imaging evaluation. The aim is to enhance the accuracy and universality of ARDS diagnosis, ultimately improving treatment and patient prognosis.

Sepsis complicated by ARDS is a leading cause of mortality in patients with sepsis in the intensive care unit (ICU). Reports indicate that annually, approximately 150,000 to 200,000 individuals worldwide succumb to sepsis complicated by ARDS. The mortality rate for patients experiencing this dual condition is estimated to be 30%–40% higher than that for patients with sepsis alone (Englert et al., 2019; Eworuke et al., 2018). Given the significant incidence and mortality associated with sepsis and ARDS (S-ARDS), establishing a reliable and effective clinical prognosis prediction model is essential. Such a model would provide intuitive, evidence-based information to assist medical professionals in identifying high-risk groups and enhancing the management of such patients.

Machine Learning (ML), a branch of artificial intelligence, enables computer systems to learn autonomously and make decisions through data analysis and pattern recognition. It is characterized by powerful data processing capabilities, automatic recognition functions, and continuous learning and optimization. In recent years, ML has become increasingly important in the development of clinical prognosis prediction models. For instance, Pappada SM et al. created a machine learning model for the early identification of ICU-acquired sepsis, achieving specificity and sensitivity rates of 83.8% and 73.3%, respectively (Pappada et al., 2024). Additionally, Fan Z et al. utilized machine learning techniques to develop a clinical prognosis model for patients with sepsis complicated by acute kidney injury, successfully validating it externally and achieving favorable clinical prediction outcomes (Fan et al., 2023).

In the realm of clinical prognostic model research for patients with sepsis and ARDS, although Mu S et al. have developed a prognostic model using data from the MIMIC-III database and the Berlin definition of ARDS, there remains a notable lack in research focusing on the clinical characteristics, prognosis, risk factor identification, and model development for sepsis patients with ARDS based on the latest global definition of ARDS.

The Critical Care Medical Marketplace (MIMIC) is a comprehensive and publicly accessible database that includes extensive information on over 190,000 patients treated at the Beth Israel Deaconess Medical Center from 2008 to 2019. This database encompasses a wide range of data, including demographic details, vital signs, laboratory test results, imaging reports, prescriptions, and clinical outcomes. It serves as a robust foundation for researching and developing clinical prognosis prediction models specifically for sepsis patients with ARDS based on the new global definition.

Therefore, this study aims to identify patients with sepsis complicated with ARDS using the new global definition from the MIMIC database, and collect their clinical characteristics, identify risk factors that affect the clinical prognosis of this population, and develop a clinical prognosis prediction model. In summary, the main contributions of this study are as follows: (1) we constructed an interpretable machine learning model to predict 28-day ICU mortality among patients with sepsis-related ARDS based on the new 2023 global definition; (2) we validated the model on an external cohort from a different hospital to demonstrate generalizability; (3) we adopted a nested cross-validation framework and SHAP analysis to ensure model robustness and interpretability; (4) we included mild ARDS patients who received only supplemental oxygen to align with the inclusive spirit of the new definition, thus improving early recognition and clinical applicability.

## 2 Methods

### 2.1 Study design and data sources

We utilize the Medical Information Mart for Intensive Care (MIMIC) database as our primary data source, specifically version MIMIC-IV 2.2. Although MIMIC-IV version 3.1 was released after our initial data extraction, we found that the Note module, which includes critical radiology and clinical notes required for ARDS diagnosis under the new global definition, had not been updated. To ensure consistency and completeness of diagnostic data, we retained version 2.2 for our study. This open-access intensive care database comprises clinical data from over 190,000 patients and 450,000 hospitalizations documented at the Beth Israel Deaconess Medical Center between 2008 and 2019, which includes approximately 70,000 ICU admissions. The MIMIC-IV database contains a wealth of information, including patient demographic details, codes from both the 9th and 10th editions of the International Classification of Diseases (ICD-9 and ICD-10), vital signs, laboratory test results, imaging studies, real-time physiological monitoring data from the ICU, and records of clinical outcomes. Importantly, all personal identifying information of patients in the database is anonymized and kept strictly confidential. Accessing and extracting data from this database necessitates approval from the relevant review committee at MIT.

We extracted data on sepsis patients with ARDS who met both the Berlin definition and the updated global definition from our database. As we all known, sepsis is defined as a disorder of the host response to infection, which leads to life-threatening multi-organ dysfunction. Consequently, the primary criteria for identifying sepsis patients in our database include clinical evidence of infection or a high suspicion of infection, along with a Sequential Organ Failure Assessment (SOFA) score of ≥2 (Singer et al., 2016).

The diagnosis of ARDS according to the Berlin definition is based on the following criteria: 1. The onset of ARDS should occur within 1 week following the onset of known clinical abnormalities or new respiratory symptoms; 2. Chest X-rays or CT scans must reveal bilateral lung infiltrates or edema, while ruling out the effects of pleural effusion or acute heart failure; 3. Mechanical ventilation is required, with a positive end-expiratory pressure (PEEP) ≥ 5 cm H_2_O and Oxygenation index (PaO2/FIO2)≤ 300 mmHg (Ranieri et al., 2012).

The new global definition of ARDS (Matthay et al., 2024) builds upon the Berlin definition, incorporating the following diagnostic criteria: 1. The onset should occur within 1 week of identified risk factors or the emergence of new or worsening respiratory symptoms, characterized by acute exacerbation or deterioration of hypoxemic respiratory failure; 2. Chest imaging must indicate bilateral lung infiltrates or edema, excluding cardiogenic pulmonary edema; 3. ARDS is classified under different ventilation states as follows: (1) Non-intubation ARDS is defined by an oxygen flow ≥30 L/min using high-flow nasal cannula (HFNC), or PEEP ≥5 cm H_2_O when using non-invasive ventilation (NIV) or continuous positive airway pressure (CPAP); (2) Intubation ARDS follows the criteria of the Berlin definition; (3) In resource-limited environments, ARDS can be diagnosed based solely on oxygen therapy, without the necessity of specific respiratory support devices such as PEEP or defined oxygen flow rates. Under these conditions, SpO2 ≤ 97% and SpO2/FiO2 ≤ 315 are considered necessary for diagnosing ARDS (Matthay et al., 2024). Although the new global definition of ARDS introduced diagnostic criteria for resource-limited settings—specifically allowing diagnosis based on supplemental oxygen therapy—this criterion was still applied in our study using the MIMIC-IV dataset from Beth Israel Deaconess Medical Center, a tertiary academic hospital. This is because, in clinical reality, even in such high-resource settings, some ICU patients may initially present with mild ARDS and receive only oxygen therapy due to adequate respiratory function. These cases, although not meeting criteria for mechanical ventilation or non-invasive support, are still eligible for ARDS diagnosis under the new global definition. Including such patients allows earlier detection of ARDS and enhances the model’s generalizability and clinical utility.

We extracted patients and their clinical data diagnosed with sepsis complicated with ARDS under the two diagnostic criteria mentioned above from the MIMIC database. We then analyzed the differences in clinical characteristics, disease severity assessments, and mortality rates between the patient groups defined by two definitions above. Furthermore, we employed machine learning techniques to predict the 28-day ICU mortality rate for sepsis patients with ARDS under the latest definition, and analyzed possible risk factors that may affect clinical prognosis.

### 2.2 Data extraction

We initially employed Structured Query Language (SQL) to retrieve and extract raw data from the MIMIC-IV database using Navicat Premium software (version 16.3.8). This data included essential clinical information about patients, laboratory test results, imaging examinations, clinical comorbidities, critical care records, advanced life support therapy details, and clinical prognosis information.

**FIGURE 1:**
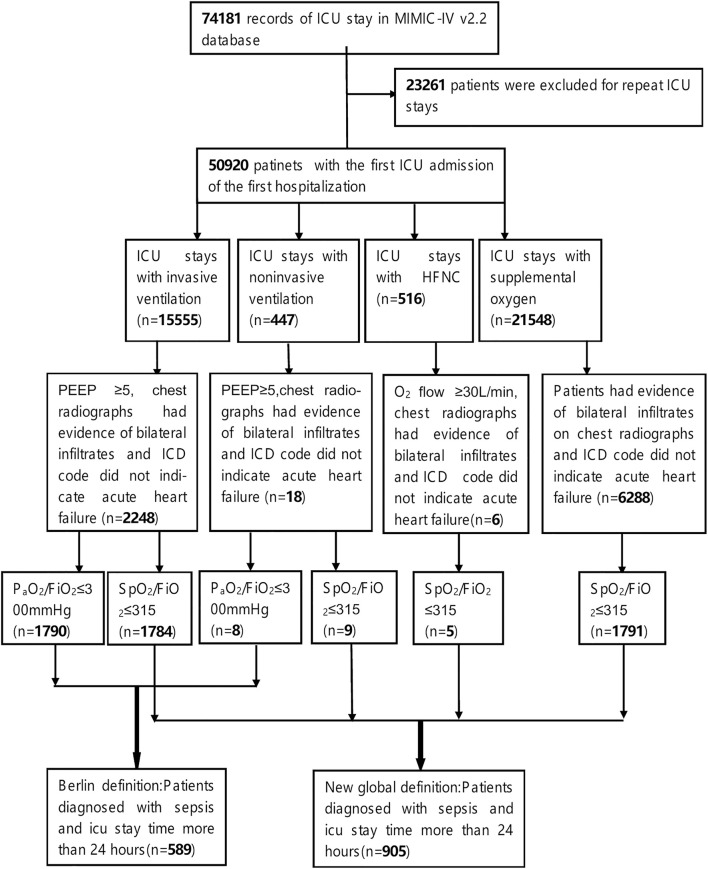
Flowchart of screening.

For this study, we included patients who met the following criteria: 1. They were experiencing their first admission to the ICU; 2. Their ICU stay exceeded 24 h; 3. They were over 18 years old at the time of admission; 4. They were diagnosed with sepsis within 24 h of admission, in accordance with the Sepsis-3.0 diagnostic criteria. To identify sepsis patients in the MIMIC-IV database, we utilized ICD-9 codes (78,552, 99,591, and 99,592), ICD-10 codes (R65.20 and R65.21), and the SOFA score recorded within the first 24 h of ICU admission; 5. Moreover, the patients were also diagnosed with Acute Respiratory Distress Syndrome (ARDS) within 24 h of admission, based on the Berlin definition or the new global definition. Detailed diagnostic criteria can be referenced in the definitions and the data extraction process illustrated in Figure 1. To ensure that the ARDS cases included in our study were induced by sepsis, we required that both the diagnosis of sepsis and ARDS occurred within the first 24 h of ICU admission. Sepsis was identified using ICD-9/10 codes (e.g., 78,552, R65.20) and a SOFA score ≥2, indicating organ dysfunction due to infection. Non-infectious causes of ARDS—such as trauma, aspiration, or pancreatitis—were excluded by design through this definition. ARDS was diagnosed using SpO_2_/FiO_2_ or PaO_2_/FiO_2_ indices and chest imaging findings recorded in the same 24-h window.

Regarding the extraction process of ARDS patients that meets the Berlin definition and the new global definition, we referred to the open-source code by Qian F et al., which includes extracting: 1. The initial ventilation treatment status of patients upon ICU admission; 2. Results from pulmonary imaging (chest X-ray or chest CT), specifically textual information indicating bilateral pulmonary edema, such as “bilateral infiltration” and “edema”; 3. PaO2/FiO2 and SpO2/FiO2 (Qian et al., 2024). Reasonable modifications were made to certain codes, for instance, we defined the PaO2/FiO2 and SpO2/FiO2 as the worst values recorded within the first 24 h of ICU admission for patients under initial ventilation treatment. If the duration of initial ventilation treatment was less than 24 h, the worst value during that treatment period was considered for the diagnostic criteria. Additionally, we employed ICD codes “428” and “I50” along with their lower-level codes to identify and exclude cases of acute cardiogenic pulmonary edema.

The data we extracted encompasses the following key elements:1. Basic Clinical Information: This includes age, gender, weight, and height at the time of admission.2. Intensive Care Records: This section details the duration of ICU hospitalization and vital signs recorded within the first 24 h of admission. Key measurements include blood pressure, heart rate, respiratory rate, body temperature, blood oxygen saturation, urine output, and blood glucose levels.3. Laboratory Test Results: Within the first 24 h of ICU admission, we collected laboratory test results, including complete blood counts, liver and kidney function tests, coagulation profiles, and arterial blood gas analyses.4. Advanced Life Support Therapy: This includes information on renal replacement therapy, mechanical ventilation, and the administration of vasoactive drugs.5. Imaging Examinations: we focused on the textual descriptions of pulmonary imaging results, such as chest X-rays and chest CT scans.6. Patient Death Records: The database contains records of patient mortality, with a positive outcome defined as death occurring within 28 days of ICU admission. In the study, vital signs and laboratory test results from the intensive care records were analyzed as independent features by utilizing their maximum, minimum, and/or mean values.

We included patients with sepsis complicated by ARDS who met the criteria of the new global definition and were admitted to Xuzhou Medical University Affiliated Hospital from March 2022 to October 2024. The exclusion criteria were consistent with those used in the training cohort. Clinical data for patients in the external validation cohort were collected based on 15 features selected from the training cohort after model training. These features included admission age, average SpO2, average body temperature, average respiratory rate, average heart rate, red blood cell distribution width (RDW), presence of metastatic solid tumors, lactate levels, urine output, international normalized ratio (INR), alkaline phosphatase levels, average red blood cell volume, logistic organ dysfunction score (LODS score), presence of rheumatic diseases, and platelet count. The inclusion and exclusion criteria for the external validation cohort were identical to those applied to the MIMIC-IV cohort. Therefore, a separate flowchart was not presented to avoid redundancy.

### 2.3 Statistical analysis

In the baseline analysis section, we employed the Shapiro-Wilks test to assess the normality of the data distribution. For continuous variables that exhibited a normal distribution, we represented them using the mean and standard deviation, and compared groups using an independent sample t-test. Conversely, for continuous variables that did not adhere to a normal distribution, we used the median and interquartile range for representation and utilized the Wilcoxon rank sum test for comparisons. Categorical data is presented as counts and percentages, with comparisons made using the chi-square test. A p-value of less than 0.05 is considered statistically significant.

Based on the survival status of patients 28 days after their admission to the ICU, we categorized them into a survival group and a death group. Additionally, patients were classified into two groups: the “Berlin definition group” and the “new global definition group,” according to their alignment with the Berlin definition or the new global definition of ARDS.

**FIGURE 2:**
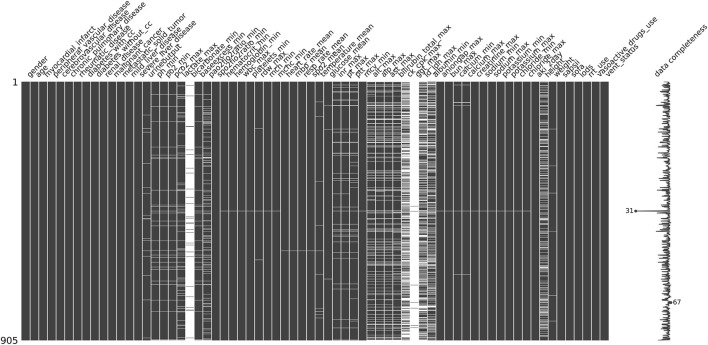
Missing value visualization: Each column represents a clinical variable, and the white lines represent missing values.

In building our machine learning models, we utilize Python version 3.11.7 along with Jupyter Notebook as our coding environment. The key packages and versions included: scikit-learn 1.4.0, miceforest 5.6.4, scikit-optimize 0.9.0, imbalanced-learn 0.12.0, SHAP 0.44.1, numpy 1.26.3, matplotlib 3.8.3. During the data preprocessing phase, illustrated in Figure 2, we employ the missing no module to visualize missing data. Each column in the visualization represents a clinical variable, with the white spaces indicating the presence of missing values. The density of the black lines in each column correlates with the number of available data points for the respective clinical variable; thus, The denser the black lines in each column, the fewer missing values for the clinical variable.

To enhance the accuracy and performance of our model predictions, we decided to exclude clinical variables with more than 30% missing values, such as bicarbonate and albumin. For the remaining missing values, we applied miceforest multiple imputation, which effectively captures complex relationships among variables by utilizing a random forest model. Through multiple iterations, the missing values are predicted in a manner that aligns with the distribution characteristics of the original dataset, thereby minimizing bias as much as possible. For continuous variables, we implement MinMaxScaler normalization to scale them appropriately, which helps eliminate dimensional effects and improves model efficiency. Additionally, we use OneHotEncoder to encode categorical variables effectively.

During the training and validation phases of our machine learning models, we evaluated several widely recognized and highly effective algorithms based on the results of feature selection using Lasso CV. These algorithms included logistic regression (LR), random forest (RF), decision tree (DT), support vector machine (SVM), lightweight gradient boosting machine (LightGBM), extreme gradient boosting machine (XGBoost), adaptive boosting machine (AdaBoost), and multilayer perceptron (MLP).

To improve the stability and generalizability of the models, we employed a repeated nested cross-validation strategy. In this approach, the outer loop involved a 5-fold cross-validation, where the dataset was randomly split into five subsets. One fold was used as the outer test set, while the remaining four served as the outer training set. Within the training set, a 10-fold inner cross-validation was conducted to perform hyperparameter tuning. This entire nested cross-validation process was repeated five times, with the dataset reshuffled before each repetition, resulting in 25 independent models per machine learning algorithm. The final performance for each algorithm was calculated as the average performance across the 25 models, which helps reduce variance due to data partitioning and ensures a more reliable model selection.

To mitigate the effects of imbalanced positive and negative outcomes on the model, we implemented the Synthetic Minority Over-sampling Technique (SMOTE) and the Tomek Link technique. These methods effectively balance the data, reduce the risk of overfitting, and enhance the model’s generalization capability. For hyperparameter optimization, we employed Bayesian Optimization to determine the optimal hyperparameter combinations. The tuned hyperparameters and search paces were as follows:

• LR: c (10^−4^ to 10^−2^)
•RF: max_depth (3–30), n_estimators (100–1,000), min_samples_split (2–10)
•DT: max_depth (3–30), min_samples_split (1–10)
•SVM: gamma (10^−4^ to 1)
•LightGBM: max_depth (3–30), num_leaves (20–200), learning_rate (0.001–0.2), n_estimators (100–1,000)
•XGBoost: n_estimators (100–1,000), colsample_bytree (0.5–1), max_depth (3–30), subsample (0.5–1)
•AdaBoost: n_estimators (50–500), learning_rate (0.01–1)
•MLP: hidden_layer_size (tuple: (50–300, 1-3 layers)).

The performance of the predictive models was assessed using various metrics, including the ROC curve, area under the curve (AUC), accuracy, sensitivity, specificity, recall, and F1 score.

In the realm of predictive model interpretation, SHAP serves as a robust tool for elucidating machine learning algorithms (Lv et al., 2023; Zhuo et al., 2023). Grounded in the Shapley value from game theory, SHAP seeks to clarify the contribution of each feature to the prediction outcomes. This approach mitigates the black box nature of machine learning models and improves their interpretability. In our study, we calculated and visualized the SHAP values for the SVC model, which demonstrated the highest predictive capability, as indicated by its AUC score.

## 3 Results

This study comprised 905 sepsis patients with ARDS who met the criteria of the new global definition (referred to as the new global definition group) and 598 sepsis patients with ARDS who met the Berlin definition (referred to as the Berlin definition group). Based on their 28-day survival status after ICU admission, the patients were categorized into two groups: a survival group and a non-survival group.

### 3.1 Baseline characteristic

Table 1 presents the distribution of patients according to varying degrees of disease severity in both the new global definition group and the Berlin definition group. In the new global definition group, there were 102 patients (11.27%) classified as mild, 278 patients (30.72%) as moderate, and 525 patients (58.01%) as severe, with a total of 336 ICU deaths (37.13%) occurring within 28 days. In contrast, the Berlin definition group consisted of 58 patients (9.85%) with mild symptoms, 208 patients (35.31%) with moderate symptoms, and 323 patients (54.84%) with severe symptoms, resulting in 228 ICU deaths (38.71%) at 28 days. Compared with the Berlin definition, the new global definition classified a slightly higher proportion of patients as severe (58.01% vs. 54.84%) and fewer as moderate (30.72% vs. 35.31%). This indicates a modest shift in severity stratification under the new definition.

**TABLE 1:** Classification of ARDS severity and 28-day ICU mortality.

Group	Total	Mild	Moderate	Severe	28-day ICU mortality
New Global Definition	905	102 (11.27%)	278 (30.72%)	525 (58.01%)	336 (37.13%)
Berlin Definition	589	58 (9.85%)	208 (35.31%)	323 (54.84%)	228 (38.71%)

Additionally, we identified 538 patients who required invasive mechanical ventilation and had both PaO2/FiO2 and SpO2/FiO2 indices by extracting cross subsets from two datasets. We then compared the 28-day ICU mortality rates among patients with varying severity levels as determined by these indices (see Table 2). A chi-square test was conducted to compare the mortality rates of the subsets, yielding a p-value of 0.597. This indicates that there was no statistically significant difference in mortality rates between the two diagnostic criteria for ARDS severity classification, which aligns with the findings reported by Qian et al. (2024).

**TABLE 2:** Comparison of mortality rates for ARDS with invasive ventilation.

Invasive Vent	Mild, n (%)	Moderate, n (%)	Severe, n (%)	p-value
ARDS with PaO2/FiO2 ratio	16 (35.56%)	55 (28.95%)	140 (46.20%)	0.597
ARDS with SpO2/FiO2 ratio	18 (30.58%)	63 (37.50%)	130 (41.80%)	

Finally, we compared the mortality rates between the invasive mechanical ventilation subgroup and the oxygen-only subgroup using the new global definition, as presented in Table 3. The chi-square test yielded a p-value of 0.439. In contrast to the findings of Qian F et al. (Qian et al., 2024), our analysis indicated that the global new definition criteria neither underestimated nor overestimated the mortality rate of sepsis patients with ARDS who received supplemental oxygen therapy.

**TABLE 3:** Comparison of mortality rates for New Global Definition Group.

Ventilation status	Mild, n (%)	Moderate, n (%)	Severe, n (%)	p-value
ARDS with invasive ventilation	21 (31.82%)	68 (38.20%)	134 (41.36%)	0.439
ARDS with oxygen	9 (25.71%)	27 (27.00%)	74 (37.56%)	

Table 4 presents the baseline characteristics of patients in the global new definition group, encompassing essential clinical data, vital signs, laboratory test results, clinical comorbidities, and records of advanced life support therapy. The overall mortality rate for this group is 37.12%. In the univariate analysis, significant differences were observed between the two groups in various factors, including age, weight, urine output, mean pulse oxygen saturation, mean arterial pressure, body temperature, pH, arterial oxygen partial pressure, lactate levels, oxygenation index, and the SpO2/FiO2 ratio, with a P-value of less than 0.001.

**TABLE 4:** Baseline characteristics of patients in New Global Difinition Group.

Variable	Survival (n = 569)	Non-survival (n = 336)	p-value
Age (year)	62.57 (50.50–73.43)	67.15 (57.51–78.87)	<0.001
Gender (%)			0.072
Male	343 (60)	182 (54)	
Female	226 (40)	154 (46)	
Height (cm)	170.00 (163.00–178.00)	168.00 (160.00–175.00)	0.003
Weight (kg)	84.00 (69.30–100.50)	78.45 (62.80–96.73)	<0.001
Urineoutput (mL)	1,320.00 (790.00–2,213.00)	890.00 (362.75–1,416.50)	<0.001
heart_rate_mean (min-1)	95.53 (83.30–106.80)	98.68 (87.85–107.58)	0.10
resp_rate_mean (min-1)	22.00 (19.15–25.15)	22.98 (19.44–26.44)	0.021
spo2_mean (%)	96.57 (95.12–97.83)	95.92 (94.26–97.84)	<0.001
mbp_mean (mmHg)	73.88 (69.44–79.55)	71.59 (66.91–76.26)	<0.001
Temperature (°C)	37.14 (36.76–37.56)	36.87 (36.50–37.34)	<0.001
ph_min	7.26 (7.18–7.34)	7.22 (7.13–7.33)	<0.001
po2_min (mmHg)	57.00 (41.00–77.00)	47.50 (38.00–68.25)	<0.001
pco2_max (mmHg)	48.00 (41.00–58.00)	48.00 (41.00–58.00)	0.87
lactate_max (mmol/L)	2.30 (1.50–3.90)	3.60 (2.00–6.50)	<0.001
baseexcess_min (mmol/L)	−6.00 (−10.00–1.00)	−7.00 (−13.00–2.00)	<0.001
aniongap_max (mmol/L)	17.00 (15.00–21.00)	20.00 (16.00–24.00)	<0.001
pao2fio2ratio_min	109.00 (72.00–172.00)	88.66 (63.75–142.13)	<0.001
spo2fio2ratio_min	131.43 (94.00–190.00)	97.00 (91.00–186.00)	<0.001
hematocrit_min (%)	30.20 (25.80–34.70)	28.00 (23.48–34.20)	<0.001
hemoglobin_min (g/dL)	9.90 (8.50–11.50)	9.20 (7.50–10.93)	<0.001
wbc_max (10^9^/L)	16.90 (10.90–24.10)	16.10 (10.25–22.65)	0.19
platelets_min (10^9^/L)	162.00 (104.00–226.00)	128.00 (61.75–210.00)	<0.001
rdw_max (%)	15.00 (14.00–16.50)	16.15 (14.70–18.43)	<0.001
mch_min (pg)	29.80 (28.40–31.20)	30.20 (28.60–31.83)	0.011
mchc_min (pg/L)	32.20 (31.20–33.30)	31.80 (30.58–33.10)	0.001
mcv_min (fl)	91.00 (86.00–95.00)	92.00 (87.00–98.00)	<0.001
alt_max (mmol/L)	36.00 (19.00–88.00)	47.00 (23.00–182.25)	<0.001
ast_max (mmol/L)	59.00 (31.00–139.00)	104.50 (40.00–382.25)	<0.001
alp_max (mmol/L)	91.00 (66.00–145.00)	110.50 (77.00–180.00)	<0.001
bilirubin_total_max (mmol/L)	1.00 (0.50–2.60)	1.20 (0.50–3.40)	0.040
creatinine_max (mmol/L)	1.50 (0.90–2.70)	1.70 (1.10–3.00)	0.014
bun_max (mmol/L)	29.00 (19.00–48.00)	38.00 (26.00–56.25)	<0.001
pt_max(s)	15.50 (13.70–19.30)	18.35 (14.50–26.93)	<0.001
ptt_max(s)	34.90 (29.80–45.70)	41.50 (31.90–63.95)	<0.001
inr_max	1.40 (1.20–1.80)	1.70 (1.30–2.50)	<0.001
glucose_mean (mmol/L)	136.67 (112.25–170.50)	136.73 (110.58–181.68)	0.76
sodium_min (mmol/L)	137.00 (133.00–140.00)	136.00 (132.00–140.00)	0.17
sodium_max (mmol/L)	140.00 (137.00–143.00)	139.00 (136.00–144.00)	0.55
calcium_min (mmol/L)	7.40 (6.90–8.00)	7.40 (6.90–8.00)	0.88
calcium_max (mmol/L)	8.20 (7.70–8.70)	8.30 (7.70–8.80)	0.13
potassium_min (mmol/L)	3.80 (3.40–4.20)	3.90 (3.40–4.40)	0.16
potassium_max (mmol/L)	4.50 (4.10–5.10)	4.80 (4.20–5.40)	<0.001
chloride_max (mmol/L)	108.00 (103.00–112.00)	106.00 (101.00–112.00)	0.24
chloride_min (mmol/L)	103.00 (98.00–107.00)	101.00 (96.00–106.00)	0.016
Sapsii	45.00 (36.00–56.00)	55.00 (46.00–66.00)	<0.001
Sofa	10.00 (7.00–13.00)	12.00 (9.00–15.00)	<0.001
Lods	8.00 (6.00–11.00)	10.00 (8.00–13.00)	<0.001
myocardial_infarct,n (%)	49 (8.6)	48 (14)	0.008
peripheral_vascular_disease, n (%)	30 (5.3)	32 (9.5)	0.014
cerebrovascular_disease, n (%)	48 (8.4)	37 (11)	0.20
chronic_pulmonTary_disease, n (%)	155 (27)	94 (28)	0.81
rheumatic_disease, n (%)	26 (4.6)	7 (2.1)	0.054
diabetes_with_cc, n (%)	35 (6.2)	17 (5.1)	0.50
diabetes_without_cc, n (%)	123 (22)	73 (22)	0.97
renTal_disease, n (%)	102 (18)	55 (16)	0.55
malignTant_cancer, n (%)	87 (15)	91 (27)	<0.001
metastatic_solid_tumor, n (%)	30 (5.3)	62 (18)	<0.001
mild_liver_disease, n (%)	123 (22)	116 (35)	<0.001
severe_liver_disease, n (%)	52 (9.1)	65 (19)	<0.001
aki_2 day, n (%)	436 (77)	298 (89)	<0.001
vasoactive_drugs_use, n (%)	389 (68)	268 (80)	<0.001
rrt_use, n (%)	35 (6.2)	34 (10)	0.030
vent_status, n (%)			0.042
Oxygen	222 (39)	110 (33)	
Highflow	1 (0.2)	1 (0.3)	
Noninvasive	0 (0)	2 (0.6)	
Invasive	346 (61)	223 (66)	

### 3.2 Feature selection

In the feature selection and screening section, we employed Lasso regression with cross-validation (Lasso CV) to evaluate various features. Lasso regression is a linear regression technique utilized for both feature selection and regularization. Its effectiveness in feature filtering primarily relies on examining the coefficients assigned to each feature within the model. Features with coefficients of zero are deemed to make no contribution to the model’s predictive power. Furthermore, Lasso regression addresses the issue of feature collinearity to some extent, with the lambda value in the regression equation governing the strength of regularization.

**FIGURE 3:**
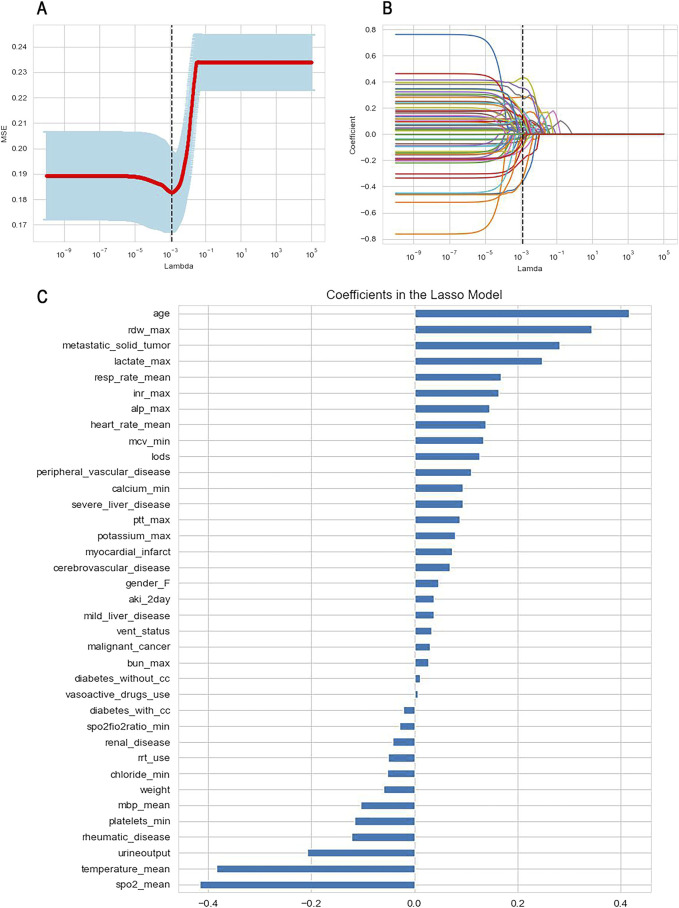
Feature selection using Lasso regression with cross-validation. **(A)** Determination of the optimal lambda value; **(B)** The variation of variable coefficients with the lambda value, the black dashed line indicates the coefficients of each variable at the optimal lambda value; **(C)** The ranking of variable coefficients.

Lasso CV integrates Lasso regression with cross-validation, automatically exploring different lambda values and utilizing cross-validation to identify the optimal alpha value. This process maximizes the balance between model complexity and fit, as illustrated in Figure 3, while also ranking the features according to their importance. We initially selected 64 candidate features based on clinical relevance identified through literature review and consultation with two intensivists, data availability in both internal and external datasets, and their accessibility within the first 24 h of ICU admission. These features covered demographics, vital signs, laboratory indicators, ventilator parameters, severity scores (e.g., SAPS II, SOFA), and comorbidities. Subsequently, we identify 37 features with non-zero coefficients using Lasso CV algorithm.

To mitigate model complexity and reduce the risk of overfitting, we selected the top 15 features based on the absolute values of their coefficients for inclusion in the machine learning model. These features comprised admission age, average SpO2, average body temperature, red blood cell distribution width (RDW), merged metastatic solid tumors, lactate levels, urine output, average respiratory rate, international normalized ratio (INR), alkaline phosphatase, average heart rate, average red blood cell volume, the Logistic Organ Dysfunction Score (LODS score), combined rheumatic system diseases, and platelet count.

### 3.3 Model performance comparison

We utilized the 15 selected features to construct machine learning models, resulting in 25 independent models corresponding to 8 different machine learning algorithms. This process included hyperparameter tuning through five iterations of 5-fold nested cross-validation, aimed at maximizing the models’ generalization ability. To comprehensively evaluate model performance, we calculated the average values and 95% confidence intervals for the area under the curve (AUC), F1 score, recall, precision, accuracy, sensitivity, and specificity of the 8 machine learning models, as detailed in Table 5.

**TABLE 5:** Prediction Performance of the 8 kinds of machine leaning algorithms.

Model	AUC	F1-score	Recall	Precision	Accuracy	Sensitivity	Specificity
Logistic Regression	0.787 (0.74–0.833)	0.644 (0.587–0.7)	0.71 (0.643–0.788)	0.592 (0.516–0.672)	0.709 (0.645–0.764)	0.71 (0.643–0.788)	0.708 (0.635–0.795)
Decision Tree	0.705 (0.664–0.768)	0.586 (0.521–0.648)	0.643 (0.504–0.808)	0.549 (0.488–0.621)	0.666 (0.615–0.717)	0.643 (0.504–0.808)	0.679 (0.519–0.807)
Random Forest	0.786 (0.742–0.842)	0.645 (0.586–0.71)	0.681 (0.57–0.776)	0.615 (0.563–0.684)	0.723 (0.682–0.772)	0.681 (0.57–0.776)	0.748 (0.696–0.796)
LightGBoost	0.755 (0.719–0.79)	0.603 (0.558–0.655)	0.616 (0.537–0.699)	0.594 (0.533–0.649)	0.7 (0.656–0.735)	0.616 (0.537–0.699)	0.749 (0.684–0.814)
AdaBoost	0.765 (0.726–0.826)	0.63 (0.583–0.696)	0.677 (0.612–0.744)	0.592 (0.527–0.677)	0.705 (0.655–0.762)	0.677 (0.612–0.744)	0.722 (0.649–0.805)
XGBoost	0.771 (0.722–0.83)	0.628 (0.559–0.676)	0.628 (0.559–0.676)	0.589 (0.529–0.647)	0.704 (0.655–0.746)	0.676 (0.573–0.773)	0.735 (0.642–0.77)
MLP	0.791 (0.741–0.841)	0.644 (0.577–0.698)	0.71 (0.634–0.776)	0.591 (0.521–0.648)	0.709 (0.65–0.756)	0.71 (0.634–0.776)	0.708 (0.633–0.779)
SVM	0.792 (0.76–0.84)	0.654 (0.588–0.711)	0.717 (0.613–0.818)	0.606 (0.554–0.658)	0.72 (0.675–0.767)	0.717 (0.613–0.818)	0.722 (0.661–0.786)

As illustrated in the table above, the SVC model demonstrates the highest AUC (95% CI) of 0.792 (95% CI: 0.76–0.84) among the eight machine learning models assessed, with the MLP model following closely behind. Additionally, other evaluation metrics, including the F1 score, recall rate, accuracy, precision, sensitivity, and specificity, indicate that the SVC model generally outperforms the other models.

**FIGURE 4:**
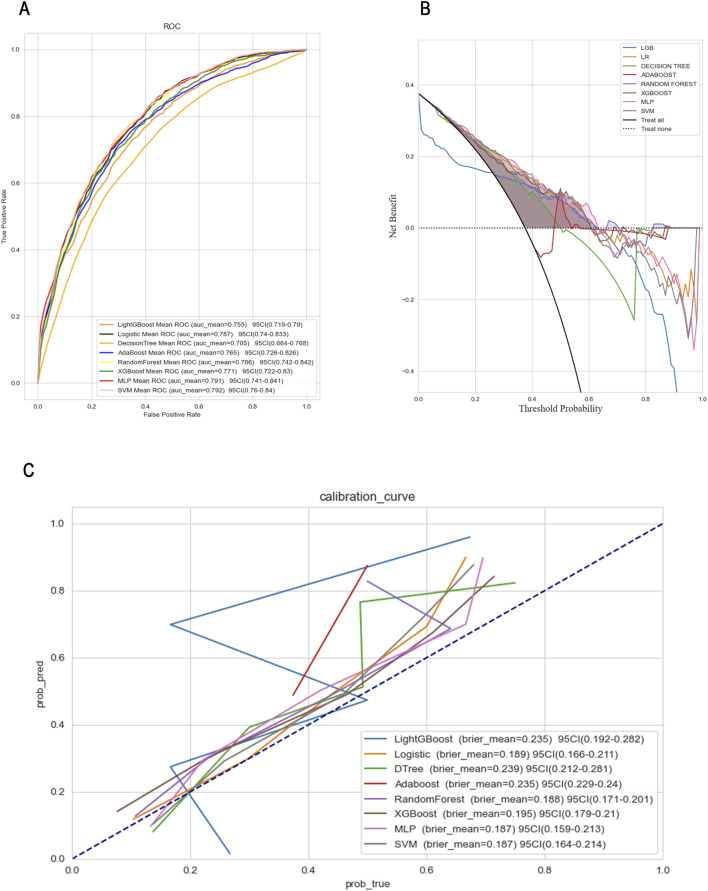
ROC **(A)**, DCA **(B)** and Calibration curves **(C)** comparison of eight models.

To further compare and visualize the performance and clinical applicability of each model, we plotted receiver operating characteristic (ROC) curves, clinical decision curves (DCA), and calibration curves (as shown in Figure 4). The ROC curve primarily assesses the classification capability of the model, illustrating its performance across various thresholds. Meanwhile, the calibration curve evaluates the accuracy of model predictions, ensuring that the outputs can be reliably interpreted as actual probabilities.

Among the eight machine learning models evaluated, SVM model exhibited relatively strong and stable performance. One possible explanation lies in the characteristics of SVM: it relies on margin maximization and distance-based computation, which makes it particularly effective when data are well-normalized and high-dimensional. Given that all features in this study were standardized prior to modeling, this may have favored SVM’s ability to find optimal separating hyperplanes. Furthermore, SVM’s capacity to handle non-linear boundaries via kernel tricks may have also contributed to its competitiveness in mortality prediction.

### 3.4 External validation

**FIGURE 5:**
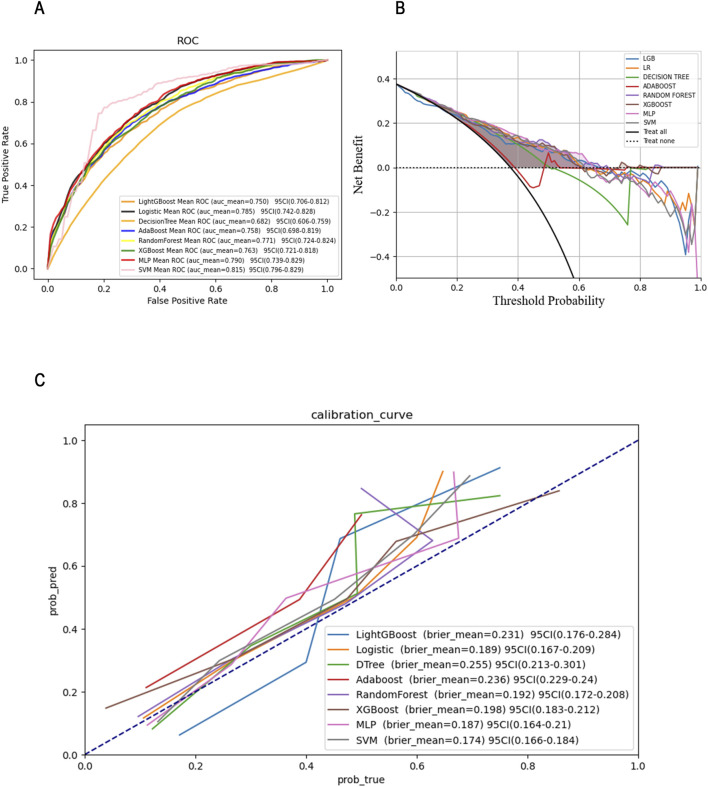
ROC **(A)**, DCA **(B)**and Calibration curves **(C)**of the SVM model in the external validation set.

We conducted an external validation of the SVC model on 100 sepsis patients with ARDS who met the criteria outlined in the new global definition and were admitted to Xuzhou Medical University Affiliated Hospital between March 2022 and October 2024 (please refer to the Supplementary Materials for a baseline comparison of the external validation cohort). Importantly, the data from the external validation cohort and the training cohort do not overlap, which enhances the assessment of the model’s generalization and predictive capabilities in real-world scenarios. The performance of the SVC model in the external validation cohort is presented in Table 6, where we observed that the model continues to demonstrate strong overall performance (Figure 5). This indicates that the predictive model developed using machine learning methods has a robust ability to forecast 28-day ICU mortality outcomes for patients with sepsis complicated by ARDS under the context of the new global definition in clinical practice.

**TABLE 6:** Prediction performance of SVC model in External validation cohort.

Model	AUC	F1-score	Recall	Precision	Accuracy	Sensitivity	Specificity
Logistic Regression	0.785 (0.742–0.828)	0.640 (0.595–0.688)	0.701 (0.631–0.785)	0.591 (0.526–0.652)	0.707 (0.655–0.746)	0.701 (0.631–0.785)	0.712 (0.642–0.786)
Decision Tree	0.682 (0.606–0.759)	0.565 (0.512–0.653)	0.624 (0.516–0.788)	0.521 (0.453–0.603)	0.646 (0.586–0.716)	0.624 (0.516–0.788)	0.659 (0.551–0.774)
Random Forest	0.772 (0.724–0.824)	0.624 (0.552–0.683)	0.655 (0.582–0.749)	0.598 (0.520–0.671)	0.706 (0.648–0.76)	0.655 (0.582–0.749)	0.736 (0.649–0.812)
LightGBoost	0.750 (0.706–0.812)	0.597 (0.538-0.675	0.605 (0.515–0.717)	0.592 (0.530–0.648)	0.698 (0.652–0.746)	0.605 (0.515–0.717)	0.736 (0.649–0.812)
AdaBoost	0.758 (0.698–0.819)	0.627 (0.571-0.691	0.680 (0.569-0.749	0.585 (0.519–0.652)	0.700 (0.648–0.754)	0.680 (0.569–0.749)	0.712 (0.623–0.772)
XGBoost	0.763 (0.721–0.818)	0.617 (0.561–0.664)	0.670 (0.605–0.728)	0.575 (0.508–0.642)	0.691 (0.636–0.738)	0.670 (0.605–0.728)	0.821 (0.611–0.786)
MLP	0.790 (0.739-0.829	0.644 (0.581–0.689)	0.706 (0.615–0.788)	0.594 (0.539–0.661)	0.710 (0.663–0.756)	0.706 (0.615–0.788)	0.713 (0.642–0.780)
SVM	0.816 (0.796–0.829)	0.726 (0.699–0.766)	0.702 (0.659–0.750)	0.758 (0.738–0.776)	0.771 (0.75–0.798)	0.702 (0.659–0.750)	0.825 (0.804–0.839)

### 3.5 Interpretability analysis

**FIGURE 6:**
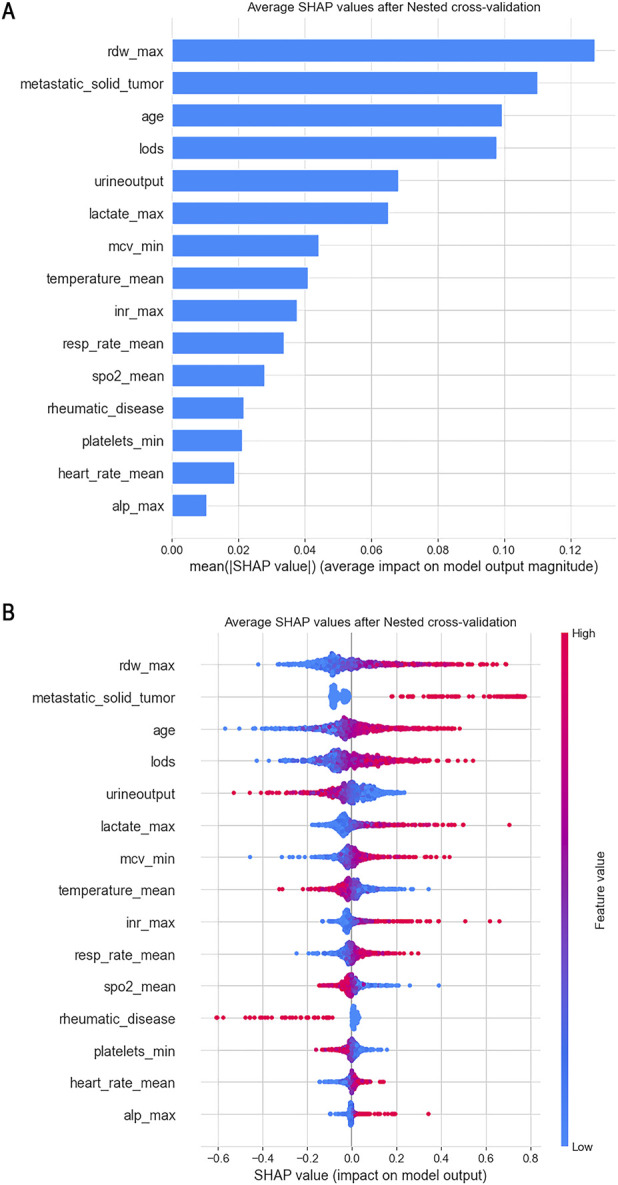
From a global perspective, we calculated the average SHAP value for each feature and used a swarm plot to display the distribution of features and SHAP values. **(A)** Plot of Features Importance; **(B)** Swarm Plot.

Given the outstanding performance of the SVC model, we computed and visualized the SHAP values to elucidate the influence of each variable on the outcomes predicted by this model. First, we examined the overall interpretability of the model by calculating the average SHAP value for each feature and ranking their importance (see Figure 6A). This analysis illustrates the overall distribution of the impact that each feature has on the model’s output.

The bee swarm plot (see Figure 6B) further displays the characteristics of data distribution by arranging numerous data points at the same horizontal position. In this plot, the X-axis represents the SHAP values of the features, while the colors indicate the magnitude of the feature values—red signifies larger feature values, and blue indicates smaller ones. Each point corresponds to a specific sample’s feature value and SHAP value; thus, the farther a point is from the X-axis, the greater its impact on the output result. Additionally, the density of points reveals the distribution of the data.

Moreover, the relationship between the color of the points (which represents the size of the feature values) and the SHAP values indicates the direction of the feature’s effect. For instance, with respect to age, larger feature values correlate with a more significant positive impact on predicting favorable outcomes, while urine output exhibits the opposite effect.

From this analysis, we can conclude that factors such as age, red blood cell distribution volume, presence of metastatic tumors, logistic organ function score, blood lactate level, international normalized ratio (INR), average red blood cell volume, average heart rate, alkaline phosphatase, and average respiratory rate are positively correlated with 28-day mortality in patients. Conversely, other indicators, including urine output and average body temperature, show a negative correlation with 28-day mortality. From a clinical perspective, adequate urine output suggests preserved renal perfusion and responsiveness to fluid resuscitation, both of which are favorable prognostic indicators in critically ill septic patients. Similarly, fever is typically a manifestation of an active inflammatory response. Previous studies have shown that moderate hyperthermia may be protective in sepsis (Beverly et al., 2016), whereas hypothermia is often linked to immune suppression and increased mortality. Therefore, these findings are biologically plausible and consistent with current understanding of sepsis pathophysiology.

**FIGURE 7:**
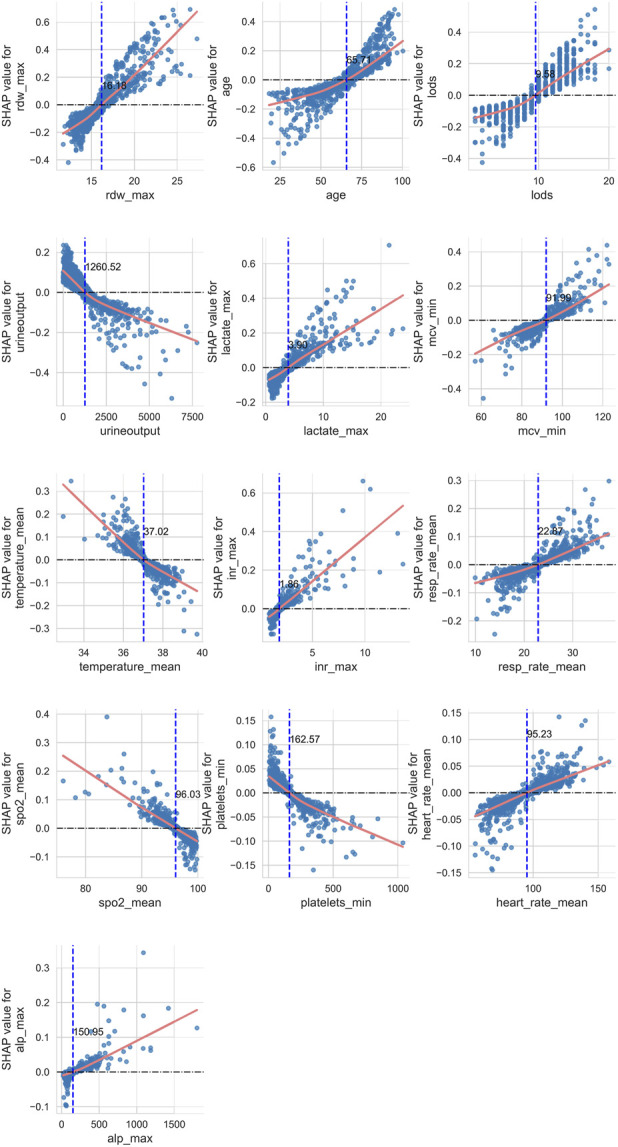
Linear or nonlinear effects of quantitative type data on prognostic outcomes.

Secondly, we investigated the complex linear and nonlinear relationships between the various features and prognostic outcomes. To achieve this, we created scatter plots of SHAP values against feature quantities for 13 quantitative data types, excluding rheumatic diseases and metastatic solid tumors among the 15 features. Additionally, we employed LOWESS fitting curves and local weighted regression to generate fitting curves, which visually represent the trend of data distribution. As depicted in Figure 7, the yellow curve indicates the fitting curve, and we highlighted the intersection point, where the SHAP value equals zero, with a blue dashed line alongside the corresponding feature value.

Using age as an example, we observed a nonlinear relationship between age and 28-day ICU mortality in patients. As age increases, its contribution to the model transitions from negative to positive, with the intersection point occurring at 65.71 years. This implies that patients older than 65.71 years are considered a risk factor for 28-day mortality.

## 4 Discussion

In this study, we employed machine learning techniques to develop and validate a predictive model for the prognosis of sepsis patients with ARDS who met the new global definition. Our model is built upon a comprehensive analysis of 64 patient features, which include basic clinical data, vital signs, laboratory test results collected within the first 24 h of ICU admission, records of advanced life support treatments, and clinical comorbidities.

To address missing data, we utilized the Miceforrest multiple imputation method. By integrating the Lasso cross-validation method with feature importance ranking, we ultimately selected 15 key features for constructing the machine learning model. During the model development phase, we employed the SMOTETomek resampling technique to balance the dataset and utilized Bayesian optimization to fine-tune the model’s hyperparameters. Additionally, nested cross-validation techniques were applied to enhance the generalization ability of various models.

Among the eight machine learning models assessed, the Support Vector Classifier (SVC) exhibited the best performance. We further elucidated the key features influencing the prognosis of sepsis patients with ARDS using SHAP values and visualization graphs. The final ROC curve and calibration curve indicate that the SVC model outperforms other models in terms of prediction accuracy. However, it is important to note that a high-performing machine learning model does not always translate to effective clinical recognition.

To evaluate and compare the clinical utility of the predictive models, we also generated DCA curves. We tested the predictive performance of the SVC model in an external validation cohort and confirmed its strong performance in real-world settings. Overall, our prognosis prediction model for sepsis complicated by ARDS, based on the SVC model, demonstrates robust performance and significant clinical applicability.

Secondly, we utilized SHAP values to elucidate the final machine learning prediction model. The feature importance map illustrates the overall influence of each feature on the predicted outcome. Meanwhile, the bee colony plot depicts the distribution of features along with the direction of their impact on the predicted results. Additionally, the combination of fitting curves and SHAP values effectively highlights the intricate relationships between individual features and outcomes, thereby facilitating more informed clinical decision-making.

In this study, we investigated the factors influencing the 28-day mortality rate of sepsis patients with ARDS who meet the new global criteria for ICU admission. From the feature importance map, we identified the five most significant factors affecting patient prognosis: red blood cell distribution width (RDW), presence of metastatic solid tumours, age, Logistic Organ Dysfunction Score (LODS), and urine output. Our results demonstrate a positive correlation between elevated RDW levels and increased 28-day mortality. RDW serves as an indicator of the variation in red blood cell volume, and numerous studies have established that a high RDW is linked to adverse outcomes in various diseases, including ARDS, cardiovascular diseases, autoimmune disorders, and malignancies (Xanthopoulos et al., 2022; Wang et al., 2019; Arkew et al., 2022; Deng et al., 2021). A recent study also highlighted the strong association between high RDW and negative outcomes in sepsis, which aligns with our findings. This correlation may stem from the inflammatory response associated with sepsis, microcirculatory dysfunction leading to shortened red blood cell lifespan, and disruptions in iron metabolism (Lorente et al., 2021).

Moreover, age plays a critical role in patient prognosis, which is easily understandable. Factors such as diminished immune function, malnutrition, and organ dysfunction can contribute to the elevated mortality risk observed in older patients suffering from sepsis combined with ARDS. The hypoxia and microcirculatory dysfunction induced by sepsis in conjunction with ARDS can result in inadequate oxygen supply to organs, leading to necrosis of renal tubular epithelial cells and subsequent renal dysfunction (Lankadeva et al., 2019). Additionally, urine output is a key indicator of microcirculatory function; thus, oliguria is a significant risk factor for mortality in patients with sepsis and ARDS.

Blood lactate levels, which indicate microcirculatory dysfunction and tissue hypoxia, are also positively correlated with adverse outcomes. The LODS score is utilized to evaluate the severity of organ dysfunction in ICU patients. While we included the SAPSII score and SOFA score in our analysis, LODS appears to have a more substantial impact on outcome prediction compared to the other two measures. Furthermore, high Mean Corpuscular Volume (MCV) is positively associated with adverse outcomes in our model. Although there is currently no literature directly linking MCV to sepsis, studies indicate that the combination of MCV and RDW can enhance the predictive accuracy for sepsis prognosis (Zhang et al., 2023).

The International Normalized Ratio (INR), which reflects coagulation function, is also closely related to poor prognoses. Similar to the findings of Schupp et al., our results indicate a positive correlation between high INR and mortality outcomes in sepsis patients (Schupp et al., 2022). The INR holds significant value in the early screening, diagnosis, and prognosis of sepsis-related coagulation disorders (Lyons et al., 2018; Zhang et al., 2021). Additionally, basic vital sign indicators—such as body temperature, blood oxygen saturation, average heart rate, and respiratory rate—are closely associated with the prognosis of sepsis patients with ARDS. Higher blood oxygen saturation indicates better preservation of lung oxygenation function. In our model, blood oxygen saturation appears to be a more effective predictor of outcomes in sepsis and ARDS patients than the oxygenation index. Although ARDS diagnosis and severity primarily depend on the oxygenation index, the continuous, cost-effective, and non-invasive nature of blood oxygen saturation measurement, along with its derived SpO2/FiO2 index, plays a crucial role in assessing ARDS severity (Wick et al., 2022).

Additionally, hypothermia was identified as a risk factor for patient mortality in this study, with the onset of hypothermia within 24 h of ICU admission potentially linked to 28-day mortality, mirroring the findings of Han et al. (2024) and Beverly et al. (2016). Lastly, we observed that the development of metastatic tumours may pose a significant risk for 28-day mortality outcomes. Research indicates that cancer patients are at a higher risk of developing sepsis, with increased mortality rates following sepsis onset (Liu M. A. et al., 2021). This heightened risk is believed to result from immune dysfunction due to the tumour itself and or cancer treatments (Williams et al., 2023). Further research is necessary to ascertain whether the notable impact of metastatic tumours in our model correlates with more severe immune dysfunction, aggressive anti-tumour therapies, or poorer nutritional status in these patients.

Conversely, our findings suggest that rheumatic diseases may act as protective factors in sepsis combined with ARDS. However, the relationship between rheumatic diseases and sepsis remains unclear. For instance, Li H et al. found in observational studies that rheumatic diseases did not correlate with an increased 28-day mortality rate in sepsis patients, except for rheumatoid arthritis, which showed a strong association with sepsis onset (Li et al., 2023). It is possible that our findings are influenced by biases related to the small sample size included in our study.

However, our research does have certain limitations. Firstly, the patient data in the database we utilized for model training primarily originated from Western countries, which differs significantly from our external validation cohort. Secondly, we focused solely on commonly used clinical data for model construction and did not perform a more comprehensive analysis of the database, potentially leading to the omission of some critical details. Finally, our study is observational and retrospective, which may introduce potential errors or biases. Nevertheless, our model demonstrated strong predictive performance in the external validation cohort.

## 5 Conclusion

In summary, machine learning methods serve as reliable tools for predicting the prognosis of sepsis patients with ARDS. Considering the current global definition of ARDS, we have refined our machine learning clinical prediction model specifically for this patient group. Additionally, we will employ model explanatory techniques to interpret the underlying information of the SVC model. This approach has the potential to significantly enhance clinical practice, assisting clinicians in developing precise, personalized treatments aimed at maximizing the survival rates of sepsis patients with ARDS.

## Data Availability

The data analyzed in this study is subject to the following licenses/restrictions: Hospital policy restrictions. Requests to access these datasets should be directed to zhaonjdoc@163.com.
